# Analysis of 4 cases of children with false-positive results of novel coronavirus-specific antibody

**DOI:** 10.1186/s12887-022-03425-9

**Published:** 2022-06-28

**Authors:** Shuzhi Dai, Jingjing Li, Jing Li, Long Li, Lin Shi, Ling Cao, Xuemei Zhong, Weijie Liu, Ying Wang, Lijuan Ma

**Affiliations:** 1 Department of Clinical Laboratory, Children’s Hospital, Capital Institute of Pediatrics, No.2 Yabao Road, Chaoyang District, Beijing, 100020 China; 2 Department of General Surgery, Children’s Hospital, Capital Institute of Pediatrics, Beijing, China; 3 Department of Cardiovascular Medicine, Children’s Hospital, Capital Institute of Pediatrics, Beijing, China; 4 Department of Respiration, Children’s Hospital, Capital Institute of Pediatrics, Beijing, China; 5 Department of Gastroenterology, Children’s Hospital, Capital Institute of Pediatrics, Beijing, China

**Keywords:** Novel coronavirus infection, Colloidal gold antibody detection method, False-positive, Mass spectrometry

## Abstract

**Background:**

This study attempts to explore the influencing factors and solutions of the colloidal gold method for novel coronavirus (2019-nCoV)-specific IgM/IgG antibody detection, summarize the clinical experience and perfect the examination process, improving the application value of antibody detection in COVID-19 diagnosis.

**Methods:**

A total of 13,329 peripheral whole blood/plasma/serum samples were obtained for COVID-19 screening from children who visited the Children's Hospital of the Capital Institute of Pediatrics outpatient clinic from April 22, 2020, to November 30, 2020. The colloidal gold method was adopted for 2019-nCoV-specific IgM/IgG antibody detection. The virus nucleic acid test results, clinical records, and serum protein fingerprint results of antibody-positive patients were collected.

**Results:**

All samples were examined using the colloidal gold method with two 2019-nCoV-specific IgM/IgG antibody detection kits. Four patients were tested single antibody-positive using both kits. The details were as follows: two cases of IgM ( +) and IgG (-) using plasma and serum separately, two cases of IgM (-) and IgG ( +) using serum and whole blood. The protein fingerprinting results and nucleic acid tests of 2019-nCoV antibodies were negative in the 4 cases. Considering the epidemiological history, clinical manifestations, and test results, these 4 children were ruled out for 2019-nCoV infection.

**Conclusions:**

When the colloidal gold method was used to detect 2019-nCoV-specific IgM/IgG antibodies, it was important to ascertain the test results as precisely as possible. Specimen type and patient history may interfere with the diagnosis.

## Background

As of the beginning of 2022, the novel coronavirus named 2019 novel coronavirus (2019-nCoV) by the WHO remains a global pandemic [1–3]. Pneumonia caused by the virus was called novel coronavirus pneumonia (COVID-19). Patients with severe 2019-nCoV infection may have difficulty breathing, multiple organ failure, or even death, which seriously threatens public health and safety [4, 5]. In the "Novel Coronavirus Pneumonia Diagnosis and Treatment Plan (Trial Eighth Edition)" issued by the General Office of the National Health and Health Commission, the etiology and serological diagnostic standards for suspected cases are outlined in Articles 3 and 4 as follows: "Positive 2019-nCoV-specific IgM/IgG antibody results" and "2019-nCoV-specific IgG antibody change from negative to positive or the IgG antibody titer in the convalescent phase increases fourfold or more than that in the acute phase." [6]. However, factors including specimen types, non-standardized specimen collection, transportation, varied infection duration, and individual discrepancies can lead to false-negative results in nucleic acid testing, affecting the disease diagnosis and treatment [7, 8]. The abovementioned problems carry substantial risk, especially for preventing and controlling infectious diseases. Specific antibody detection could effectively compensate for nucleic acid detection deficiencies. Among them, the colloidal gold method is simple and has minimal requirements for the detection environment [9, 10].

Moreover, the colloidal gold test is fast and highly specific, which has a certain value in rapidly identifying clinically suspected COVID‑19 cases with negative nucleic acid results [11]. In this study, the colloidal gold method was adopted to detect 2019-nCoV-specific IgM/IgG antibodies in 13,329 patients in our hospital. The clinical records and relevant test results of the double-positive patients were analyzed to explore possible factors of false-positive results. Therefore, we aimed to effectively identify false-positive results and perfect the examination process to provide a factual basis for clinical or differential diagnosis.

## Patients and methods

### Patients selection

A total of 13,329 children who visited the outpatient clinic of the Children's Hospital of the Capital Institute of Pediatrics (No.2 Yabao Road, Chaoyang District, Beijing, China) from April 22, 2020, to November 30, 2020, were subjected to 2019-nCoV-specific IgM/IgG antibody detection. There were 8083 males and 5246 females, ranging in age from 1 day to 17 years old.

### Methods

#### The colloidal gold testing kit

From April 22, 2020, to July 21, 2020, whole blood samples (EDTA anticoagulation tube) were used for antibody detection. Moreover, plasma (derived from whole blood samples centrifuged at 3500 rpm/min for 10 min in an EDTA anticoagulation tube)/serum sample (derived from whole blood samples centrifuged at 3500 rpm/min for 10 min in a vacuum blood collection tube containing separating gel, after standing for 30 min at room temperature) was used for antibody detection from July 22, 2020, to November 30, 2020. All samples were examined with a 2019-nCoV IgM/IgG antibody detection kit (colloidal gold method).

Detection method: (1) Remove the test card from the kit and add 20 μL of a venous whole blood sample or 10 μL of serum/plasma sample into the circular holes of the IgM/IgG antibody detection reagent. (2) Later, add 2 drops (80μL) of sample diluent and stand at room temperature. (3) Observe the results within 15 min. Samples with positive IgM/IgG antibody results are reexamined with another 2019-nCoV IgM/IgG antibody detection kit (B kit, colloidal gold method). Detection method: (1) Defrost the sample-required reagents to room temperature. (2) Take the test card and lay it flat on a dry surface. (3) Add 20μL venous whole blood sample or 10μL serum/plasma sample to the IgM/IgG test card holes (S). (4) Then, add 2 drops (100μL) of sample diluent vertically into the sample holes. Observe the results within 15 min.

The technical parameters of the 2019-nCoV-specific IgM/IgG antibody detection kit used in this study (kit A and B) are shown in Table 1.

**Table 1:** Technical parameters of kit A and B kit

Name	Company	Targeted antigen	Detected antibody	Sensitivity	Specificity
A. 2019-nCoVIgM /IgG antibody detection kit (colloidal gold method)	INNOVITA Biotechnology Co., Ltd. (Tangshan)	Fused segment of N protein and S protein	Combined detection of IgM and IgG	87.3%	100%
B.2019-nCoV IgM /IgG antibody detection kit (colloidal gold method)	ZHU HAI LIVZON DIAGNOSTICS INC.,	Fused segment of N protein and S protein	Separate detection of IgM and IgG	IgM: 79.0%, IgG: 84.3%, Combined: 90.6%	IgM: 99.7%, IgG: 99.4%, combined: 99.2%

2019 nCoV denotes 2019 novel coronavirus, IgM denotes immunoglobulin M, and IgG denotes immunoglobulin G. The above information about each antibody detection kit is obtained from the clinical trial results stated in its instructions

#### Protein fingerprint detection (mass spectrometry)

The Ebio ReaderTM 3700 M time-of-flight mass spectrometry system and supporting reagents produced by Beijing East–West Analytical Instruments Co., Ltd. were adopted. The 4 antibody-positive samples in our cohort were subjected to protein fingerprinting (mass spectrometry) detection.

## Results

### Antibody detection

Among the 13,329 samples, 0.22% were tested single-positive by one of the detection kits. At the same time, unanimously positive by two detection kits: a case of IgM (-) IgG ( +) whole blood sample, a case of IgM ( +) and IgG (-) plasma sample, a case of IgM ( +) IgG (-) serum sample, and ta case of IgM (-) and IgG ( +) serum sample. The details are listed in Table 2. Combining mass spectrometry, epidemiological history, and clinical manifestations for a comprehensive analysis, all other populations included in the study were free of COVID-19 infection.

**Table 2:** Clinical data of 4 cases antibody-positive samples

NO	Gender	Age	Clinical Diagnosis	Sample Type	Results
1	Male	6y + 6 m	Intussusception	Whole Blood	IgM(-), IgG( +)
2	Male	1y + 2 m	Mucocutaneous lymph node syndrome	Plasma	IgM( +), IgG(-)
3	Female	4y + 1 m	Infectious mononucleosis	Serum	IgM( +), IgG(-)
4	Female	12y + 11 m	Mycoplasma pneumoniae pneumonia	Serum	IgM(-), IgG( +)

### Mass spectrometry

**Fig. 1:**
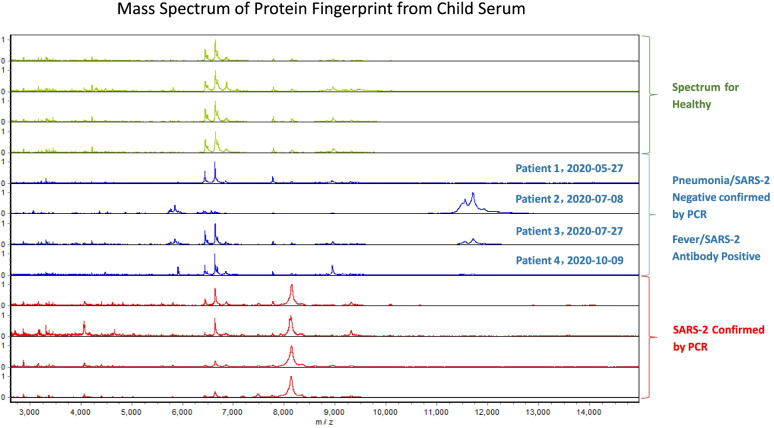
Comparison of protein fingerprinting of COVID-19 positive samples and healthy controls. The red spectrum represents the COVID-19 positive sample. The blue spectrum represents the nucleic acid-negative and antibody-positive samples from children with fever. The green spectrum represents the samples from healthy children

The above comparison showed that the 4 patients were all negative in mass spectrometry (Fig. 1). Further combined with epidemiological history, clinical manifestations, and 2019-nCoV nucleic acid negative results, 2019-nCoV infection was eventually ruled out. These 4 antibody-positive samples were all false-positive, with a false-positive rate of 0.03%.

### Cases trace-back of 2019-nCoV antibody-positive patients

The false-positive reasons for the 4 cases require further exploration.

#### Case 1

A male child, 6.5 years old, was admitted to the General Surgery Department with the chief complaint of "abdominal pain accompanied with vomiting after surgery for intussusception". Intussusception reduction was performed on the child at the local hospital 12 days before admission. The 1.5U red blood cell transfusion was administered twice because of anemia after the operation. EDTA anticoagulated whole blood samples were obtained and used for the 2019-nCoV IgM/IgG antibody detection with the help of reagents A and B, respectively. The results from both kits were IgM (-) and IgG ( +). The protein mass spectrometry result returned negative. Later, the patient’s plasma sample was reexamined on May 29, 2020, and the test results of both kits were IgM (-) and IgG (-). Our analysis ascribed the false-positive result to antibody production after blood transfusion.

#### Case 2

A male child, 14 months old, was admitted to the Department of cardiology for a chief complaint of "fever for 7 days, rash for 3 days, red eye for 2 days" and was clinically diagnosed with mucocutaneous lymph node syndrome. EDTA anticoagulated plasma samples were examined for 2019-nCoV IgM/IgG antibodies using reagents A and B, respectively. The results were both IgM ( +) and IgG (-). Meanwhile, the child’s coagulation function test showed a significantly elevated fibrinogen (FiB) concentration of 5.57 g/L. However, after the removal of FiB, the results were all IgM (-) and IgG (-). On July 8, 2020, the protein mass spectrometry result returned negative. After treatment, the patient's plasma sample was reexamined. Moreover, the results from both kits were IgM (-) and IgG (-). Also, the FiB level was 2.82 g/L on the same day. Our analysis believed an increased FiB level could cause false-positive results.

#### Case 3

A female child, 4 years and 1-month-old was diagnosed with infectious mononucleosis due to "fever, rash for 6 days, nasal congestion and snoring for 4 days" in the outpatient clinic. The patient's peripheral blood serum samples were tested for 2019-nCoV IgM/IgG antibodies using reagents A and B, respectively. Furthermore, the results were all IgM ( +) and IgG (-). Moreover, the percentage of heterogeneous lymphocytes in the peripheral blood was 18%, EBV-DNA was positive, and EBV capsid antibody IgM was > 160 (positive), all of which led to the diagnosis of EB virus infection. The protein mass spectrometry result returned negative. After treatment, the patient's serum sample was reexamined, and the test results from both kits were IgM (-) and IgG (-). In our analysis, we believed the false-positive result could be caused by an elevated heterophile antibody (HA) level.

#### Case 4

A female child, 12 years and 11 months old, was hospitalized in a Beijing Children's Hospital chief complaint of "fever, cough, and wheezing". The discharged diagnoses were as follows: severe *MYCOPLASMA PNEUMONIA*, hypoxemia, and allergic rash. The patient presented to the respiratory unit with a CT showing right lower lobe pneumonia and a preliminary diagnosis of "*MYCOPLASMA PNEUMONIA*". The doctor concluded that the patient needed to be hospitalized for bronchoscopic lavage treatment. The patient's peripheral blood serum samples were tested for 2019-nCoV IgM/IgG antibodies using reagents A and B, respectively. The test results were IgM (-) and IgG ( +). At the same time, the protein mass spectrometry result returned negative. The laboratory results at admission were as follows: *MYCOPLASMA PNEUMONIA* IgM antibody positive, with an antibody titer > 1:640, abnormal blood coagulation, elevated FiB level of 4.12 g/L, increased D-dimer level of 0.68 mg/LFEU. We thought an elevated heterophile antibody level in our analysis could cause a false-positive result.

## Discussion

The immune response would be initiated during virus infection, in which cellular immunity and humoral immunity play a vital role in anti-viral therapy [12]. After infection, pathogen-specific proteins, including nucleocapsid protein (NC) and spike protein (S) of 2019-nCoV, would effectively induce humoral immune response [13], predominantly mediated by B lymphocytes. Once the body is infected, B lymphocytes can differentiate into plasmocytes and then synthesize and secrete antibodies, a kind of globulin with the immune function that can specifically bind to corresponding antigens. The IgM is the first antibody produced in the initial humoral immune response. The increase in IgM level indicates current infection, which can be used for early diagnosis of infection. Moreover, IgG is the most abundant antibody primarily produced during the second immune response. A study has shown that 2019-nCoV-specific IgM antibodies are produced 5 to 7 days after infection, while IgG antibodies are secreted 10 to 15 days after infection [14].

Immunocolloidal gold is a diagnostic technique that uses colloidal gold as a tracer marker for qualitatively detecting biological macromolecules. The National Medical Products Administration of China approved the detection kits A and B used in this study. The study had found that the detection rates of IgM and IgG in the serum samples of 189 suspected patients with negative 2019-nCoV nucleic acid results were 59.8 and 52.9%, respectively, with an IgM/IgG combined detection rate as high as 66.1% [15]. A study by Xu et al. [16] shows that 16 patients were positive for 2019-nCoV IgM antibodies among 19 patients with negative nucleic acid test results but were diagnosed with COVID-19 based on clinical symptoms, with a positive rate of 84.21%. Moreover, there were 18 patients positive for 2019-nCoV IgG antibodies, with a positive rate reaching 94.74%. These results indicated that antibody detection could effectively compensate for the missed diagnosis of nucleic acid test, thus playing a vital role in the timely diagnosis and treatment of COVID-19 [16]. An in-depth study revealed that in the early stage of infection, the sensitivity of the antibody detection exceeds that of the nucleic acid test. The sensitivity of the combined nucleic acid test and serum antibody detection can reach as high as 99.4%, which is 32.3% higher than that of nucleic acid detection alone [17]. Also, higher levels of IgM and IgG antibodies can be detected in patients with severe COVID-19, which are closely related to the disease phase at detection [18].

Although the 2019-nCoV-specific antibody test can compensate for the low positive rate, time-consuming, and high risk of nucleic acid testing, there are certain false-negative and false-positive results for the specific antibody test. The reasons for false negatives and false positives are mainly related to the pattern of antibody production and the inherent interference of immunological methods. Therefore, it is necessary to minimize the false-positives of IgM/IgG antibody detection from the methodological design and clinical applications to avoid misleading clinical diagnoses and the resulting medical resource waste and to effectively exert the value of specific antibody detection.

Protein fingerprinting technology comprises two parts: protein chip and time-of-flight mass spectrometry. There are over 50,000 proteins in the body; for each disease, a corresponding set of proteins is regulated and expressed. Similarly, pneumonia caused by viral infections such as the 2019‑nCoV and influenza induces protein expression. When the captured protein chip is put into the mass spectrometer, protein molecular ions produced under laser bombardment fly in the tube, generating a complete protein fingerprint spectrum. Based on proteomics and protein fingerprinting technology, differential proteins of the disease were identified and used for diagnosis and pathogenic mechanism research. Of those, protein fingerprinting technology lies at the core of this method. The employment of protein chips for serum sample purification is a key technology for the application.

In our study, the Ebio Reader 3700 fully automated time-of-flight mass spectrometry system developed by Beijing East–West Analytical Instrument Co., Ltd. was used as a platform. With the help of a weak cationic protein chip to capture specific proteins in the serum of pneumonia samples, the application program basis for rapid 2019-nCoV screening was established. According to research, the protein fingerprints of different families of 2019-nCoV (groups B and C) are similar. The accuracy of the map for 2019-nCoV positive and negative samples has reached 100%. The protein fingerprint of an asymptomatic patient was highly consistent with that of the positive map, though the nucleic acid test was negative. Moreover, the protein fingerprint of a weakly antibody-positive sample was in line with the negative map. Moreover, the clinical diagnosis of that patient was negative.

The 4 patients with positive results from both detection kits in this study were positive for a single antibody. The false-positives included antibodies cross-reaction after blood transfusion, immune antibodies cross-reaction after mycoplasma infection, and the endogenous interfering substances of FiB and HA. Clinical studies have pointed out that during blood transfusion, the risk of red blood cell sensitization increases by 1.0 to 1.5% for every 1 U of plasma or red blood cell transfusions. With multiple times of blood transfusions, the risk of production of the same antibody can be as high as 20% [19]. Meanwhile, the probability of irregular antibody production increases [20].

In case 1, the child had two red blood cell transfusions twice within two weeks. The irregular antibodies were produced after blood transfusions, which had cross-reactions with coating antigens, resulting in false-positives. After this false-positive case, we communicated with clinical physicians. We switched the specimen type from whole blood to plasma or serum, which reduced the false-positive rates caused by hemocytes to a certain extent.

In case 2, the FiB level of the patient was significantly higher than normal. However, when the FiB level returned to normal after 10 days, the antibody detection result was IgM (-) and IgG (-) at reexamination. According to other research, the FiB level would increase when the blood shows hypercoagulative status, which would interfere with coating specific antigens on colloidal gold and lead to false-positives [21].

In case 3, the child had infectious mononucleosis, and the first antibody detection was interfered with by increased HA level, leading to false-positives. The antibody test result was IgM (-) and IgG (-) at reexamination after two weeks. HA is a multispecific immunoglobulin produced after antigenic stimulation and has a weak binding capacity to a wide range of immunoglobulins of the species [22, 23]. Studies have found that HA in humans includes natural antibodies and autoantibodies. Most HAs are natural antibodies, the predominant types that interfere with antibody detection [24]. First found in the serum of infectious mononucleosis patients, HA was found in 3% to 15% of healthy patients. HA was found in 3% to 15% of healthy patients. Cross-linking these heterophile antibodies can produce a false-positive reaction [25].

In case 4, the patient had *MYCOPLASMA PNEUMONIA*. The mycoplasma IgM antibody was positive, with a titer of > 1:640. Also, the FiB level was elevated. Interestingly, the antibody was negative as detected by the chemiluminescence method. Therefore, we considered that the cross-reaction of immune antibodies caused the false-positive result after mycoplasma infection and increased the FiB level [26]. In most 2019-nCoV IgM/IgG antibody detections, S protein and/or N protein were adopted as targeted antigens. According to reports, immune cross-reaction was found between N protein and/or S protein of different coronaviruses [27], and the overall specificity of RBD and S1 antigen is superior to S and N antigens [17, 28, 29]. In the chemiluminescence assay, the sensitivity of acridinium ester-labeled neo-IgM and IgG as target antigens for coronavirus-specific antibodies was 70.24 and 96.1%, respectively; and the specificity was 96.2 and 92.41%, respectively [30].

In addition, endogenous interfering substances such as rheumatoid factors [31, 32], complement and lysozyme, and exogenous factors include hemolysis, prolonged storage time, contamination with microorganisms, incomplete coagulation, or insufficient centrifugation are also the reasons for false-positive results [33].

The false-positive samples in this study were all positive for a single antibody, with no IgM ( +) and IgG ( +) results found, suggesting that the combined detection of 2019-nCoV-specific IgM/IgG can avoid the misleading of false-positives to some extent. The sensitivity and specificity of the colloidal gold IgM/IgG antibody combined detection kit was 88.66% and 90.63%, respectively [34]. Single IgM/IgG positive is rare in clinical practice. Analysis of 58 patients who had presented with symptoms for 8–33 days showed that 94.83% of patients were positive for both IgM and IgG, while 1.72% and 3.45% of patients were only positive for IgM or IgG [34]. Moreover, flow gaging immunochromatography technology was used to detect 2019-nCoV-specific antibodies in 397 confirmed cases and 128 healthy controls. The results showed that the sensitivity of the combined detection of IgM and IgG was higher than that of single IgM/IgG detection [34]. For clinically suspected patients, the combined and dynamic detection of specific IgM/IgG antibodies can help determine whether the results of the initial antibody test were reliable, thereby avoiding false-positive results.

The corresponding improvement should be actively taken when the false-positive results of 2019-nCoV are discovered, including optimizing the inspection process, switching sample types, detailed collection of medical history, and interference analysis of certain diseases. The clinical data of the 4 cases is relatively complete. Also, the biological protein profile is used to detect the amino acid sequence of the whole proteome of 2019-nCoV. High accuracy carries certain significance for the reexamining of 2019-nCoV antibody-positive samples [35]. Nevertheless, further large-scale clinical studies are needed to confirm whether protein fingerprinting can be used as a method to examine 2019-nCoV antibody-positive samples.

## Conclusions

False-positives in IgM/IgG antibody detection are common in clinical practice. Though unavoidable, feasible measures should be further explored to reduce false-positives as much as possible. The reports of false-positives may cause unnecessary quarantine and panic and interfere with normal medical order, especially in highly infectious public health emergencies. Therefore, in clinical laboratory examination, physicians should take patients’ clinical data into full consideration, rationally optimize the process, make up for the testing deficiency and effectively identify various interference factors that could lead to false-positive results. Timely communication with doctors for clinical diagnosis, thus providing valuable test results.

## Data Availability

The datasets used and/or analyzed during the current study are available from the corresponding author on reasonable request.

## Abbreviation

2019-nCoV: 2019 Novel coronavirus

## Declarations

### Competing of interests

The authors declare no complete interests.
